# Bilateral Pulmonary Cystic Lesions with Fatal Outcome Due to Echinococcosis

**DOI:** 10.4269/ajtmh.24-0511

**Published:** 2025-02-18

**Authors:** Aman Elwadhi, Prateek Kumar Panda, Indar Kumar Sharawat

**Affiliations:** Department of Pediatrics, All India Institute of Medical Sciences, Rishikesh, India

**Figure 1.**
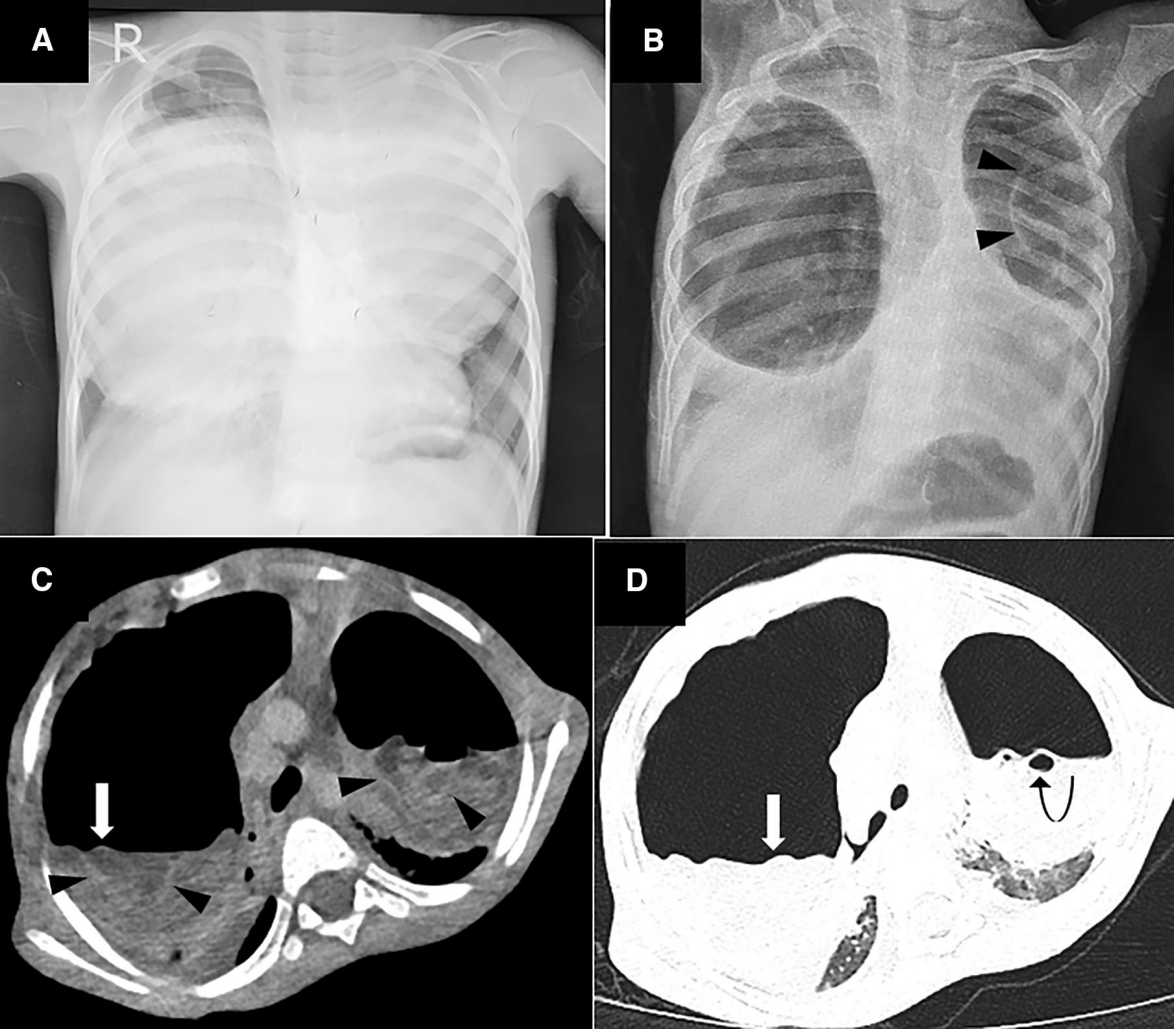
Chest radiographs and chest computed tomography (CT) scan of the child. (**A**) Initial chest radiograph showed large well-defined rounded opacities in bilateral lungs. (**B**) Repeat radiograph showed large, rounded air-containing cavities in bilateral lungs, with radio-opaque crumpled material within (Waterlily sign, arrowhead). (**C**) CT of thorax, mediastinal window, and (**D**) lung window showed well-defined bilateral cysts, with air-fluid level (arrow), with dependent wavy crumpled laminated contour, created by floating parasitic membranes (Waterlily/Camelotte sign; arrowhead) and air foci in the endocyst (air bubble sign; curved arrow).

A 4-year-old boy presented with fever, cough, and tachypnea for 20 days, worsening over the last 5 days. A chest radiograph showed bilateral pleural fluid collections and suspected empyema. He underwent thoracentesis at a community hospital, after which his respiratory distress worsened. Upon presentation, hypoxemic respiratory failure necessitated intubation and mechanical ventilation. The initial chest radiograph from the community hospital revealed large, well-defined rounded opacities in bilateral lungs (Figure 1A); a repeat radiograph showed large, rounded air-containing cavities with the water lily sign (arrowhead; Figure 1B). Computed tomography of the thorax further demonstrated well-defined bilateral cysts, with air-fluid levels (arrow), a wavy crumpled laminated contour (floating parasitic membranes; arrowhead), and foci of air (curved arrow; Figure 1C-D). These findings suggested pulmonary hydatid cyst (PHC). An ELISA showed *Echinococcus granulosus* IgG antibodies. During surgery, the patient experienced hypoxia, followed by shock, and he died during the procedure.

Most PHCs do not produce any symptoms until they rupture. Giant PHCs (with a maximum diameter of >10 cm) are more common in children because of their greater lung elasticity. Pulmonary hydatid cysts are primarily located in the lower lobes (55–70% of cases) and can sometimes be multiple (30%) and bilateral (20%).^1^ The radiological manifestations of PHCs variably reflect the lack of rupture, incomplete rupture, complete rupture, and/or super-infection. Hydatid cysts are typically composed of multiple layers, including a tough outer pericyst and a delicate inner germinal layer that produces cystic fluid and protoscolices, which may lead to daughter cysts.^2^

The early identification and prompt surgical management of pulmonary hydatid disease are essential because cyst aspiration increases the risk of rupture, secondary infection, and anaphylaxis, thereby increasing morbidity and mortality, as occurred in our case.^3^ Aspiration of a lung lesion of unknown origin should be avoided. Pulmonary hydatid disease should be considered a high-priority differential diagnosis for cystic lung lesions in endemic areas of low- and middle-income countries. Clinicians need to be aware of this condition to avoid invasive procedures and ensure prompt referral to tertiary care centers.
